# High-flow nasal cannula for acute respiratory failure: a bibliometric analysis of current trends and future directions

**DOI:** 10.3389/fmed.2026.1811474

**Published:** 2026-05-21

**Authors:** Siying Chen, Zixia Wen, Chenxi Wang, Jianhua Li, Kang Zou

**Affiliations:** 1 The First Clinical Medical College, Gannan Medical University, Ganzhou, Jiangxi, China; 2 School of Humanities and Social Sciences, Gannan Medical University, Ganzhou, Jiangxi, China; 3 Department of Critical Care Medicine, The First Affiliated Hospital of Gannan Medical University, Ganzhou, Jiangxi, China

**Keywords:** acute respiratory failure, bibliometric analysis, COVID-19, high-flow nasal cannula, research trends

## Abstract

**Background:**

High-flow nasal cannula (HFNC) oxygen therapy has emerged as a pivotal non-invasive respiratory support modality for acute respiratory failure (ARF), fundamentally transforming critical care practice. Despite substantial growth in HFNC research and clinical significance, no comprehensive bibliometric analysis has systematically examined the knowledge structure, research trends, and emerging frontiers in this rapidly evolving field.

**Methods:**

A comprehensive bibliometric analysis was conducted using the Web of Science Core Collection (WOSCC), supplemented by PubMed and Scopus for literature retrieval, covering publications from January 2015 to December 2025. Search terms included combinations of “acute respiratory failure,” “respiratory failure,” “ARF,” “high-flow nasal oxygen,” “high-flow oxygen,” and “HFNC” in titles and abstracts. Data were analyzed using three visualization tools: CiteSpace (version 6.4.R1), VOSviewer (version 1.6.20), and Bibliometrix in R (version 4.5.1) to examine publication trends, country collaborations, author networks, journal distributions, co-citation patterns, and keyword evolution.

**Results:**

A total of 1,477 publications were identified, with annual output peaking at 256 in 2022, reflecting intensified research during the COVID-19 pandemic. The United States led in productivity (*n* = 139), while France achieved the highest citation impact (TC = 7,726). Keyword and co-citation clustering revealed evolving research foci from foundational respiratory support (2015–2017) to pandemic-driven applications (2019–2021) and emerging artificial intelligence (AI) integration (2022–2025). Nine distinct research clusters were identified, with recent emphasis on chronic obstructive pulmonary disease, reintubation, and advanced respiratory technologies.

**Conclusion:**

The findings highlight HFNC's successful transition from experimental therapy to evidence-based standard care, with future trends indicating movement toward personalized medicine approaches and technology-enhanced respiratory care delivery systems.

## 1 Introduction

High-flow nasal cannula (HFNC) oxygen therapy has emerged as a pivotal non-invasive respiratory support modality for patients with acute respiratory failure (ARF), fundamentally transforming clinical practice in critical care settings (1). HFNC delivers heated and humidified oxygen at flow rates up to 60 L/min, providing physiological benefits including improved oxygenation, reduced work of breathing, and generation of modest positive end-expiratory pressure (2). Recent clinical guidelines from the American College of Physicians and European Respiratory Society have established evidence-based recommendations for HFNC use, highlighting its efficacy in reducing intubation rates compared to conventional oxygen therapy (3). The 2024 SRLF-SFMU consensus conference further emphasized HFNC's role in acute hypoxemic respiratory failure management, particularly following the COVID-19 pandemic (4). Despite growing clinical adoption, machine learning studies indicate that predicting HFNC failure remains challenging, highlighting the need for continued research optimization (5, 6).

Bibliometric analysis has become an indispensable tool for mapping scientific landscapes and identifying emerging research trends across diverse academic disciplines. The exponential growth of bibliometric studies, with publications increasing from 51 in 2000 to over 3,400 in 2022, reflects the increasing emphasis on evaluating research quality and impact through quantitative methods (7). This systematic approach enables researchers to rapidly screen, classify, and organize extensive literature information, facilitating discovery of new research fields and identification of knowledge gaps (8). Modern bibliometric analysis employs sophisticated visualization tools such as CiteSpace and VOSviewer, which provide comprehensive insights into research collaboration networks, citation patterns, and thematic evolution (9). Recent studies demonstrate that while bibliometric software tools are making noteworthy contributions to research, their visibility and standardized application remain areas for improvement (10).

Despite the substantial growth in HFNC research and its clinical significance, no comprehensive bibliometric analysis has systematically examined the knowledge structure, research trends, and emerging frontiers in this field. Therefore, this study aims to conduct a comprehensive bibliometric analysis of HFNC research in ARF from 2015 to 2025, utilizing advanced visualization tools including CiteSpace, VOSviewer, and Bibliometrix. Knowledge mapping and emerging trends in the application of high-flow nasal cannula oxygen in acute respiratory failure: a visual analysis based on bibliometric tools.

## 2 Materials and methods

### 2.1 Data sources and search strategy

For this research, searched three internationally recognized academic databases: the Web of Science Core Collection (WOSCC), PubMed and Scopus. These platforms were selected for their extensive coverage of peer-reviewed publications across a wide range of disciplines, as well as their robust indexing and citation tracking features, which are particularly advantageous for bibliometric studies. The search was limited to original research articles and review papers published in English, with a publication timeframe spanning from January 1, 2015, to December 31, 2025. To minimize potential biases arising from database updates or incomplete indexing, all relevant records were retrieved and reviewed in a single comprehensive search cycle. The specific search queries employed are detailed below: (TI = (“acute respiratory failure” OR “respiratory failure” OR ARF) OR AB = (“acute respiratory failure” OR “respiratory failure” OR ARF)) AND (TI = (“high-flow nasal oxygen” OR “High-flow oxygen” OR HFNC) OR AB = (“high-flow nasal oxygen” OR “High-flow oxygen” OR HFNC)) in WOSCC. ((“acute respiratory failure” [Title/Abstract] OR “respiratory failure” [Title/Abstract] OR ARF [Title/Abstract]) AND (“high-flow nasal oxygen” [Title/Abstract] OR “High-flow oxygen” [Title/Abstract] OR HFNC [Title/Abstract])) in PubMed. (TITLE (“acute respiratory failure” OR “respiratory failure” OR arf) OR ABS (“acute respiratory failure” OR “respiratory failure” OR ARF)) AND (TITLE (“high-flow nasal oxygen” OR “High-flow oxygen” OR HFNC) OR ABS (“high-flow nasal oxygen” OR “High-flow oxygen” OR HFNC)) in Scopus.

**Figure 1:**
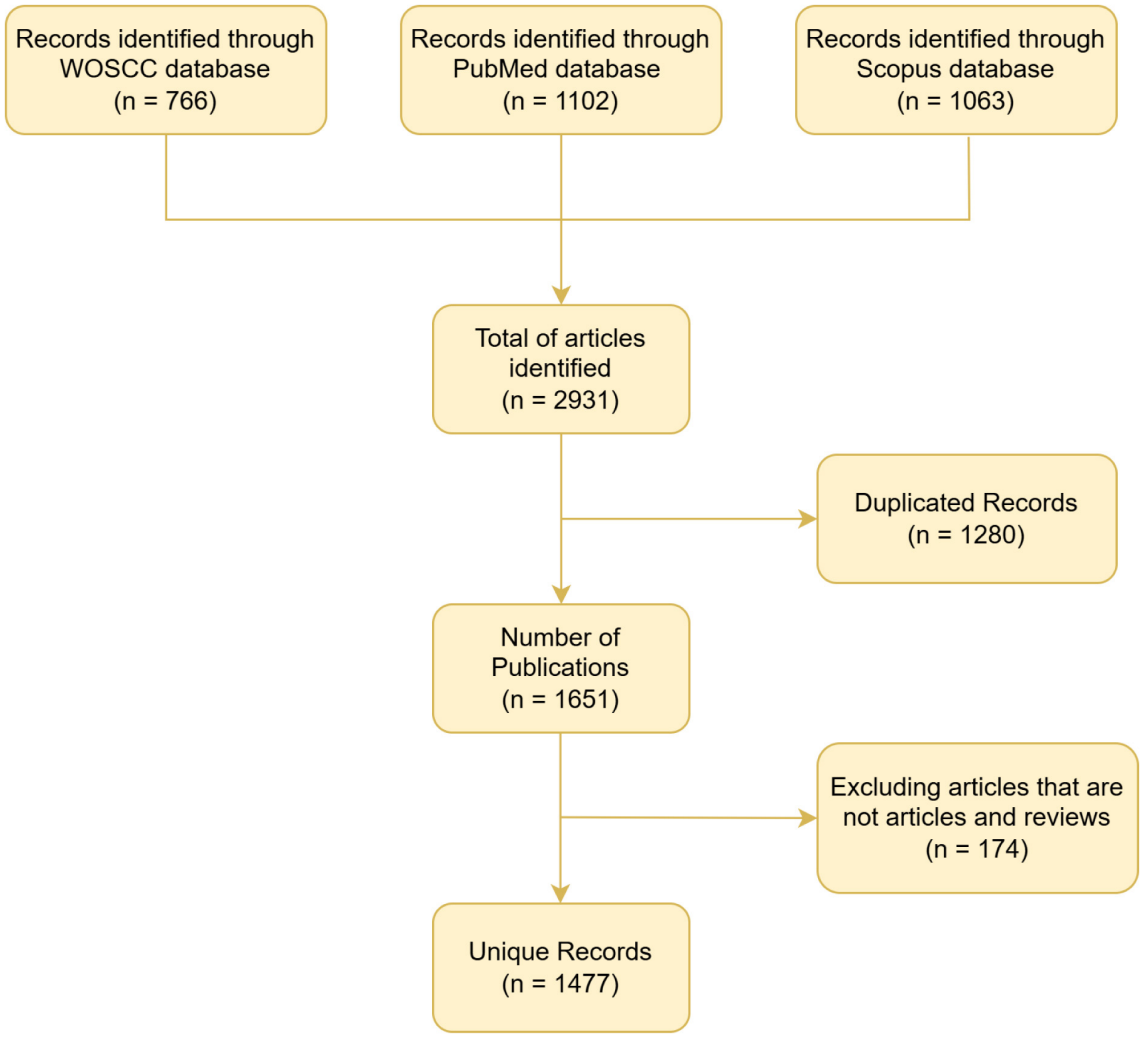
Step-by-step literature review process.

Following the application of predefined inclusion criteria, a total of 1,477 records were retrieved, exported in plain-text format, and archived for downstream processing. Duplicate and anomalous entries were rigorously eliminated, and retracted publications were excluded by sequentially deploying the deduplication algorithms embedded in CiteSpace (v. 6.4.R1) and EndNote X9. A detailed flow diagram summarizing each step of the document selection pipeline is provided in Figure 1. To mitigate field-loss inherent to the merged WOSCC-PubMed-Scopus dataset, keyword co-occurrence analyses were executed on the consolidated corpus, whereas all remaining bibliometric indicators were computed exclusively from the WOSCC subset.

### 2.2 Data analysis and visualization

The data extracted from the database comprised article counts, citation frequencies, countries/regions, authors, journals, institutions, and reference keywords (11, 12). These data were subsequently imported into three analytical tools: Bibliometrix, CiteSpace (version 6.4.R1, developed by Chaomei Chen at Drexel University, China), and VOSviewer (version 1.6.20, developed by Van Eck and Waltman at the Centre for Science and Technology Studies, the Netherlands) for visual and quantitative analysis. CiteSpace was employed to perform reference and keyword citation burst analysis, as well as journal double-map overlays, to identify emerging research directions and developmental trajectories (13, 14). VOSviewer was used to construct and visualize co-authorship and co-occurrence networks among authors, countries, and keywords. Additionally, Bibliometrix—an R-based open-source tool developed by Massimo Aria and Corrado Cuccurullo—was utilized for tracking trends in author keywords and mapping thematic evolution using R version 4.5.1 (15).

## 3 Results

### 3.1 Annual trend of publications

**Figure 2:**
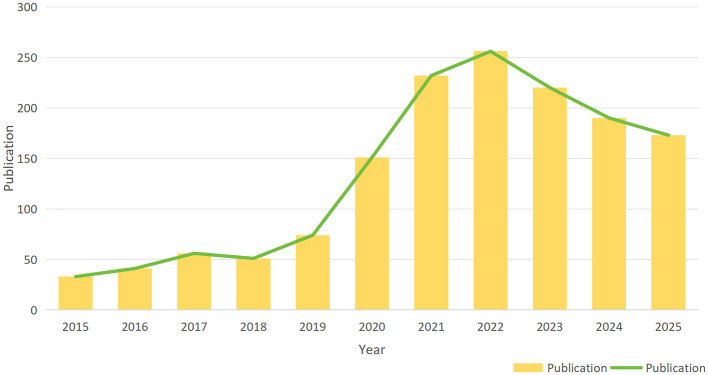
Yearly publication trends for HFNC in ARF studies.

A total of 1,477 publications were identified between 2015 and 2025. Publication volume serves as a partial indicator of research trends within the field. As illustrated in Figure 2, the annual number of publications on HFNC in ARF increased markedly from 33 in 2015 to a peak of 256 in 2022, followed by a gradual decline to 173 by 2025. This pattern suggests a period of intensified investigative focus between 2017 and 2022, with subsequent activity stabilizing near pre-surge levels.

### 3.2 Analysis of countries/regions and institutions

**Figure 3:**
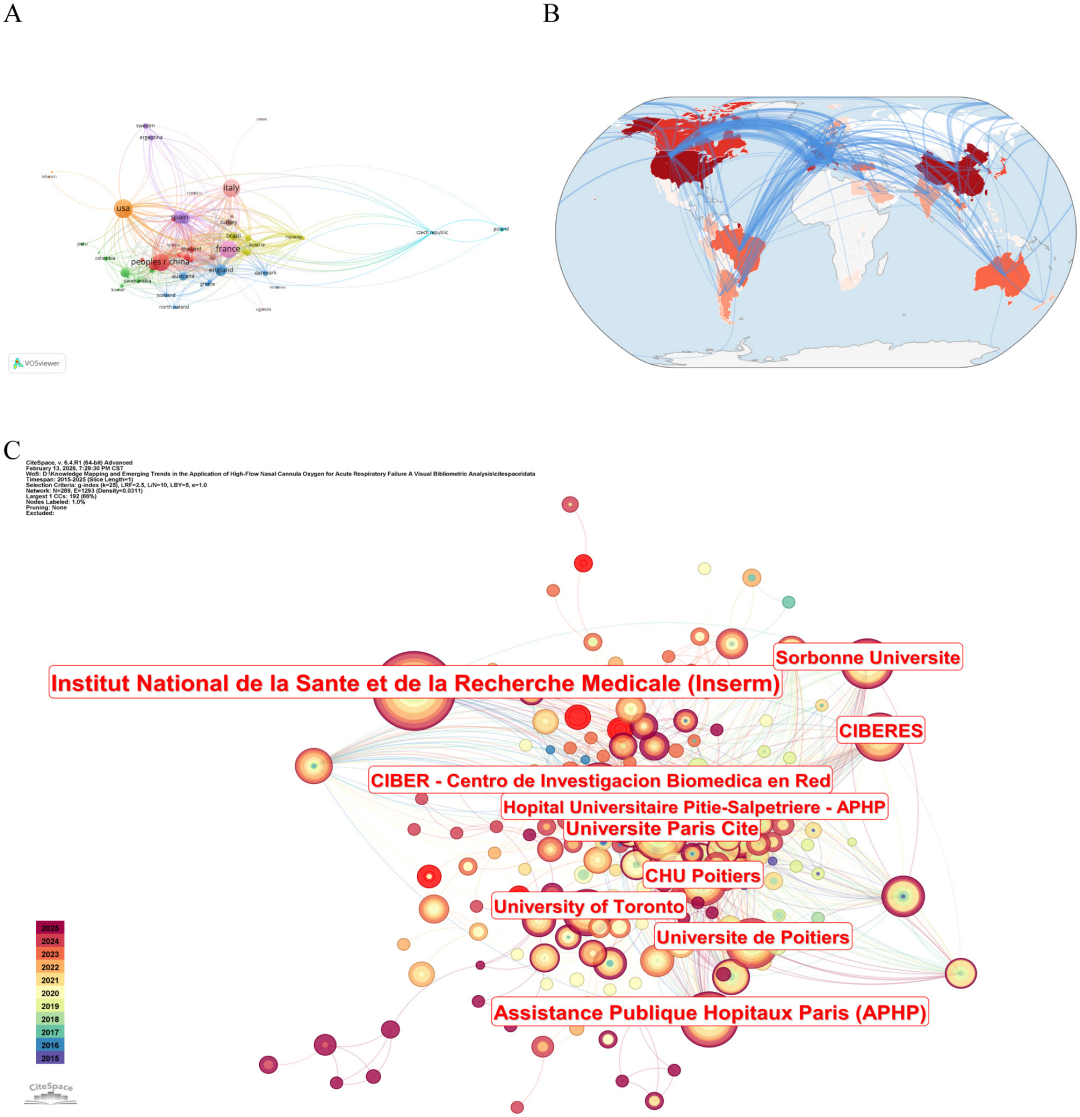
Global and organizational contributions to HFNC research in ARF. **(A)** International collaborative authorship network. **(B)** Geographic distribution of publication counts across countries/regions. **(C)** Institutional co-occurrence network analysis in the field of HFNC in ARF research.

The United States produced the highest number of publications (*N* = 139), whereas France, despite ranking fourth in output (*N* = 123), achieved the highest total citation count (TC = 7,726), indicating particularly high research influence (Table 1). A country co-authorship network was generated using VOSviewer based on collaborative relationships between nations. Countries were included if they had published at least one article; seven without collaborative links were excluded, resulting in a final set of 59 countries (Figure 3A). In the network map, node size corresponds to publication volume, and connecting lines represent collaborative strength, with thicker lines indicating stronger partnerships. The United States had the largest number of publications, while France exhibited the most extensive collaborations. Figure 3B illustrates the global distribution of publications, with North America, Europe, and Asia emerging as the most productive regions. Figure 3C presents an institutional co-occurrence map generated with CiteSpace (time span: 2015–2025; time slice: 1 year; *g*-index: *k* = 25). Node size reflects citation frequency, and nodes with purple rings indicate high betweenness centrality (≥0.1), denoting key influential institutions. Table 2 lists the top 10 countries ranked by betweenness centrality. The Autonomous University of Barcelona (Spain) recorded the highest centrality (0.09), exceeding that of more prolific French institutions. The top three institutions by betweenness centrality were the Autonomous University of Barcelona (0.09), Centre Hospitalier Universitaire d'Angers (0.07), and CIBERES (0.07). Four of the leading institutions were from France.

**Table 1:** Leading 10 nations by publication count.

Rank	Country	No. of documents	Total citations
1	The United States	139	3,740
2	Italy	128	5,299
3	France	123	7,726
4	Peoples R China	120	2,834
5	Spain	71	4,112
6	England	57	2,320
7	Canada	54	4,370
8	South Korea	38	1,265
9	Japan	35	831
10	Australia	26	1,110

**Table 2:** Ten organizations with the highest intermediary centrality scores.

Rank	Affiliation	Freq	Degree	Centrality	Country
1	Autonomous University of Barcelona	20	40	0.09	Spain
2	Centre Hospitalier Universitaire d'Angers	18	44	0.07	France
3	CIBERES	40	41	0.07	Spain
4	CIBER—Centro de Investigacion Biomedica en Red	40	41	0.07	Spain
5	Copenhagen University Hospital	3	19	0.07	Denmark
6	Assistance Publique Hopitaux Paris (APHP)	58	45	0.06	France
7	Hopital Universitaire Saint-Louis—APHP	24	37	0.06	France
8	Hopital Universitaire Pitie-Salpetriere—APHP	28	37	0.06	France
9	McMaster University	13	14	0.06	Canada
10	Universite Paris Cite	44	39	0.05	France

### 3.3 Author analysis

**Figure 4:**
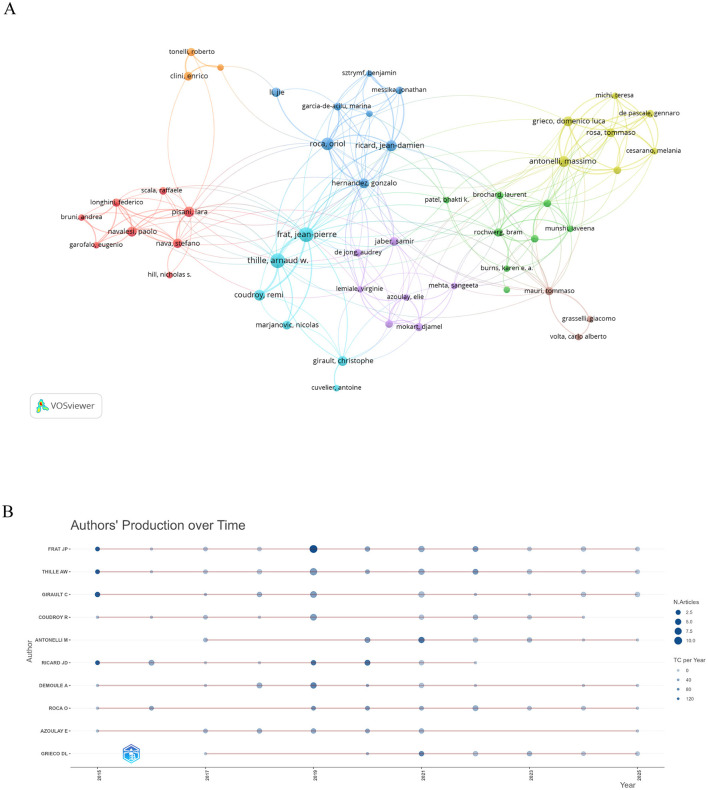
Scholarly contributors in HFNC for ARF research. **(A)** Co-authorship network mapping for 60 investigators publishing five or more joint papers. **(B)** Publishing activity timeline for the top 10 most active researchers.

A total of 60 authors who had co-authored at least five publications were included, and their collaborative networks were visualized (Figure 4A). The top 10 authors ranked by publication output are presented in Table 3. Frat JP led in publication count, citation numbers, *H*-index, and *G*-index, demonstrating considerable scholarly influence in the field. Figure 4B illustrates the temporal trends in publication and citation counts for these leading authors. Notably, a 2015 article by Frat JP entitled “High-Flow Oxygen Through Nasal Cannula in Acute Hypoxemic Respiratory Failure” received the highest number of citations (TC = 1,576).

**Table 3:** Leading 10 authors by publication volume.

Rank	Author	NP	TC	*H*-index	*G*-index
1	Frat JP	36	4,100	16	36
2	Ricard JD	19	3,864	15	19
3	Thille AW	34	3,249	16	34
4	Girault C	26	2,759	13	26
5	Brochard L	10	2,421	7	10
6	Ragot S	12	2,231	10	12
7	Nseir S	11	2,219	10	11
8	Mercat A	3	2,152	3	3
9	Robert R	9	2,106	9	9
10	Prat G	7	2,029	6	7

### 3.4 Journal analysis

**Figure 5:**
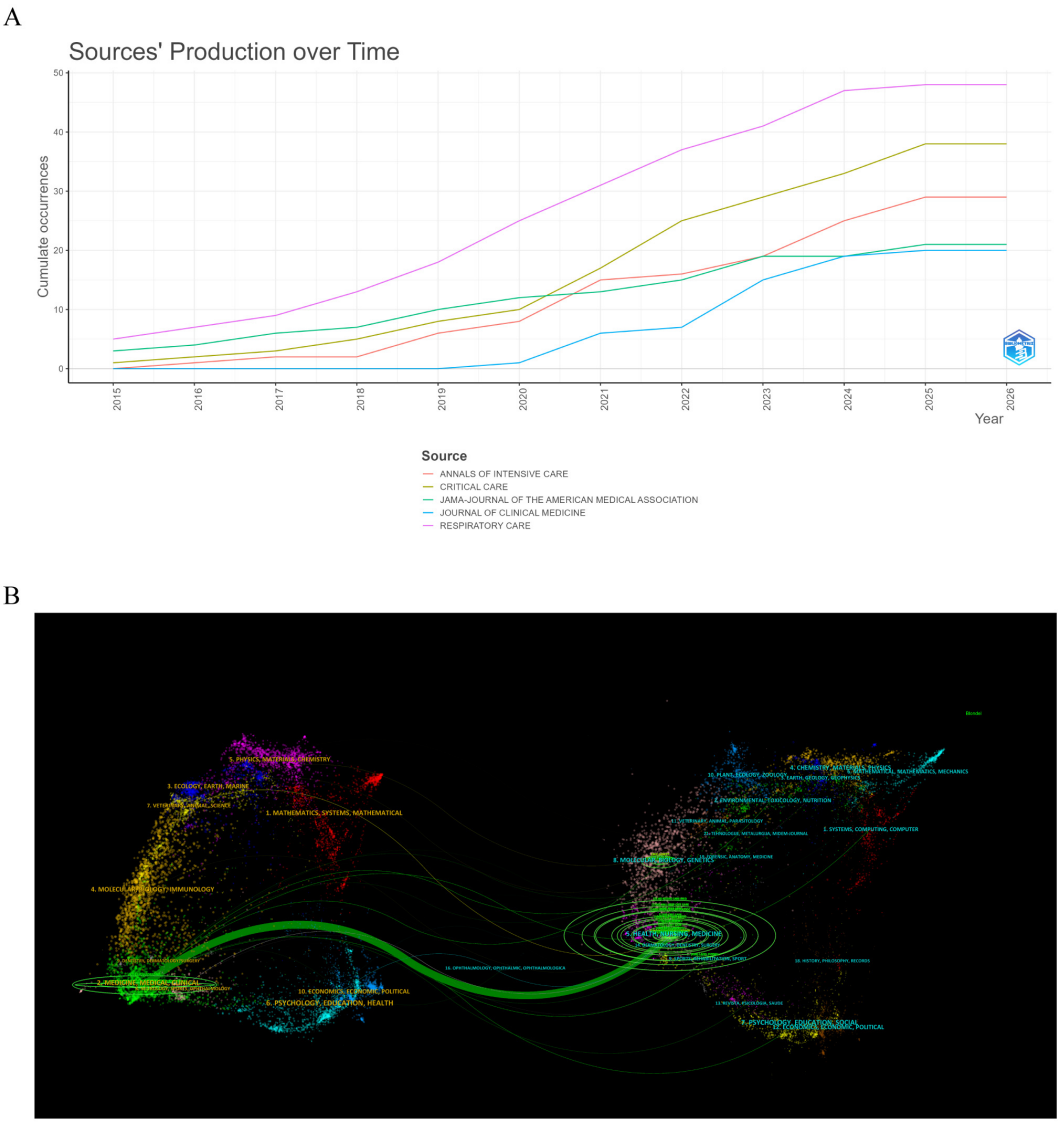
Journal analysis and knowledge base in HFNC for ARF research. **(A)** Temporal trends in publication output from core journals. **(B)** Dual-map overlay of journal citations.

Based on the analysis of core journals, *Respiratory Care* published the most articles (*n* = 48), while *JAMA-Journal of the American Medical Association*—the journal with an IF of 55.0—led in citations (*n* = 2,578) and total link strength (391). *Critical Care* also showed a notably high total link strength (437), underscoring the centrality of critical care in this research domain (Figure 5A and Table 4). The dual-map overlay technique, established by Chen et al., and Zhao et al. (16, 17), was employed to trace interdisciplinary knowledge dissemination within high-flow nasal oxygen in critical illness for the period 2000–2025 (Figure 5B). In the generated map, the left and right hemispheres correspond to citing and cited journals, respectively, while the colored arcs represent citation trajectories between these journal clusters. Field labels annotate the major disciplinary areas involved in these knowledge transfer processes.

**Table 4:** Ten most prolific journals in terms of published content.

Rank	Journal	Documents	Citations	Total link strength	IF (2025)
1	*Respiratory Care*	48	1,734	418	2.1
2	*Critical Care*	38	1,679	437	9.3
3	*Annals of Intensive Care*	29	809	287	5.5
4	*JAMA-Journal of the American Medical Association*	21	2,578	391	55.0
5	*Journal of Clinical Medicine*	20	202	136	2.9
6	*BMJ Open*	19	117	80	2.3
7	*Frontiers in Medicine*	18	140	116	3.0
8	*European Respiratory Journal*	17	732	109	16.6
9	*Angewandte Chemie-International Edition*	17	1,264	64	16.9
10	*Frontiers in Pharmacology*	17	634	59	4.8

### 3.5 Keyword analysis

**Figure 6:**
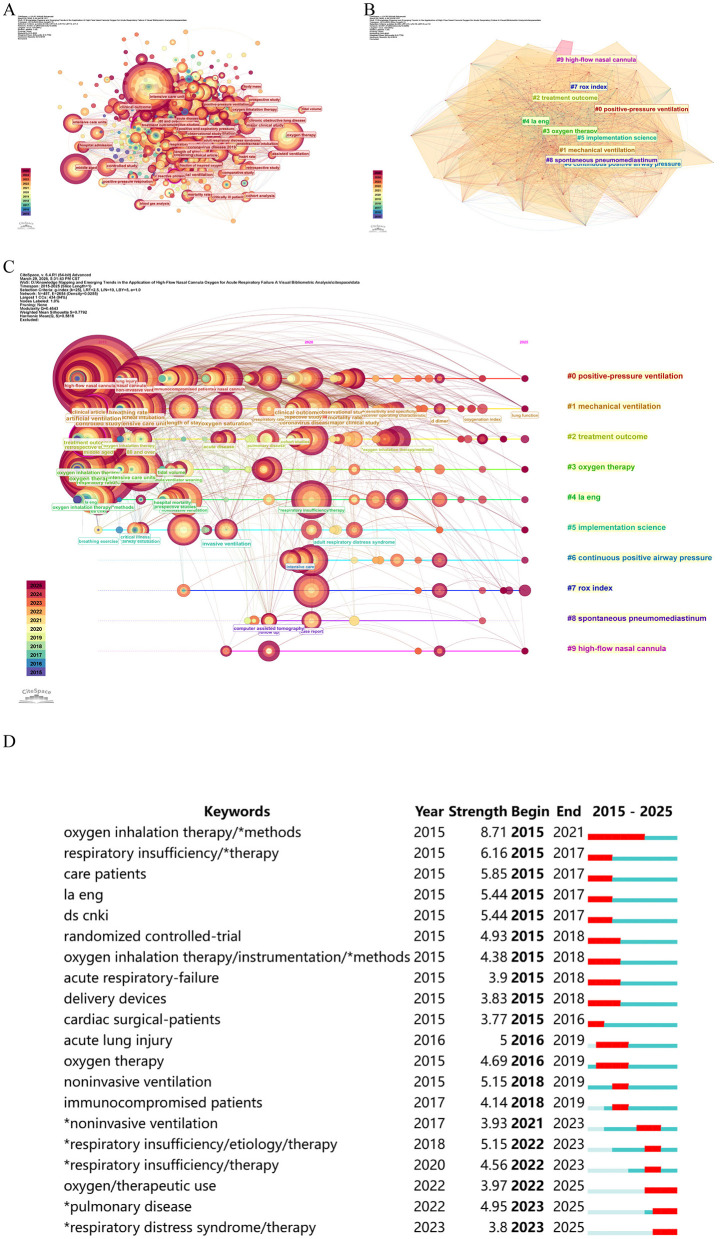
Keyword analysis and conceptual evolution in the application of HFNC in ARF. **(A)** Co-occurrence network of high-frequency keywords. **(B)** Thematic clustering of research foci. **(C)** Temporal evolution of major research themes. **(D)** Top 20 keywords with the strongest citation bursts. ^*^Indicates keywords with significant citation burst strength.

Keyword co-occurrence analysis was conducted using keywords as nodes. Among the 457 keywords identified, the 10 most frequent were: *non-invasive ventilation* (*n* = 343), *respiratory failure* (*n* = 251), *high-flow nasal cannula* (*n* = 207), *acute respiratory failure* (*n* = 167), *oxygen therapy* (*n* = 164), *mechanical ventilation* (*n* = 143), *oxygen inhalation therapy* (*n* = 136), *middle aged* (*n* = 132), *intensive care unit* (*n* = 107) and *non-invasive ventilation* (*n* = 95). In the resulting co-occurrence network (Figure 6A), the node size is proportional to keyword frequency.

Cluster analysis was performed to identify major research themes and their evolution. Keywords were categorized into 10 distinct clusters, representing predominant research foci: #0 positive-pressure ventilation; #1 mechanical ventilation; #2 treatment outcome; #3 oxygen therapy; #4 la eng; #5 implementation science; #6 continuous positive airway pressure; #7 rox index; #8 spontaneous pneumomediastinum and #9 high-flow nasal cannula (Figure 6B). This clustering delineates the conceptual structure and developmental trajectories within the field.

Diachronic analysis from 2015 to 2025 reveals the evolution of research hotspots. The network exhibited a modularity (*Q*) of 0.4643 and an average silhouette (*S*) value of 0.7792. A *Q*-value > 0.3 suggests significant cluster structure, and an *S* value > 0.5 indicates reasonable clustering credibility (18). Timeline visualization identified the most recently active research fronts as Cluster #0 (positive-pressure ventilation), #1 (mechanical ventilation), and #2 (treatment outcome; Figure 6C).

To further evaluate temporal trends, a thematic evolution analysis was conducted. Research focus evolved markedly from 2015 to 2025: early publications (2015–2017) emphasized cardiac surgery patients and basic respiratory care, while research intensity peaked between 2019 and 2021, coinciding with the COVID-19 pandemic, with dominant themes including mechanical ventilation, non-invasive support, and intensive care management. Recent trends (2022–2025) reflect growing interest in performance optimization and advanced respiratory technologies (Figure 6D).

### 3.6 Co-citation bursts and citation bursts

**Figure 7:**
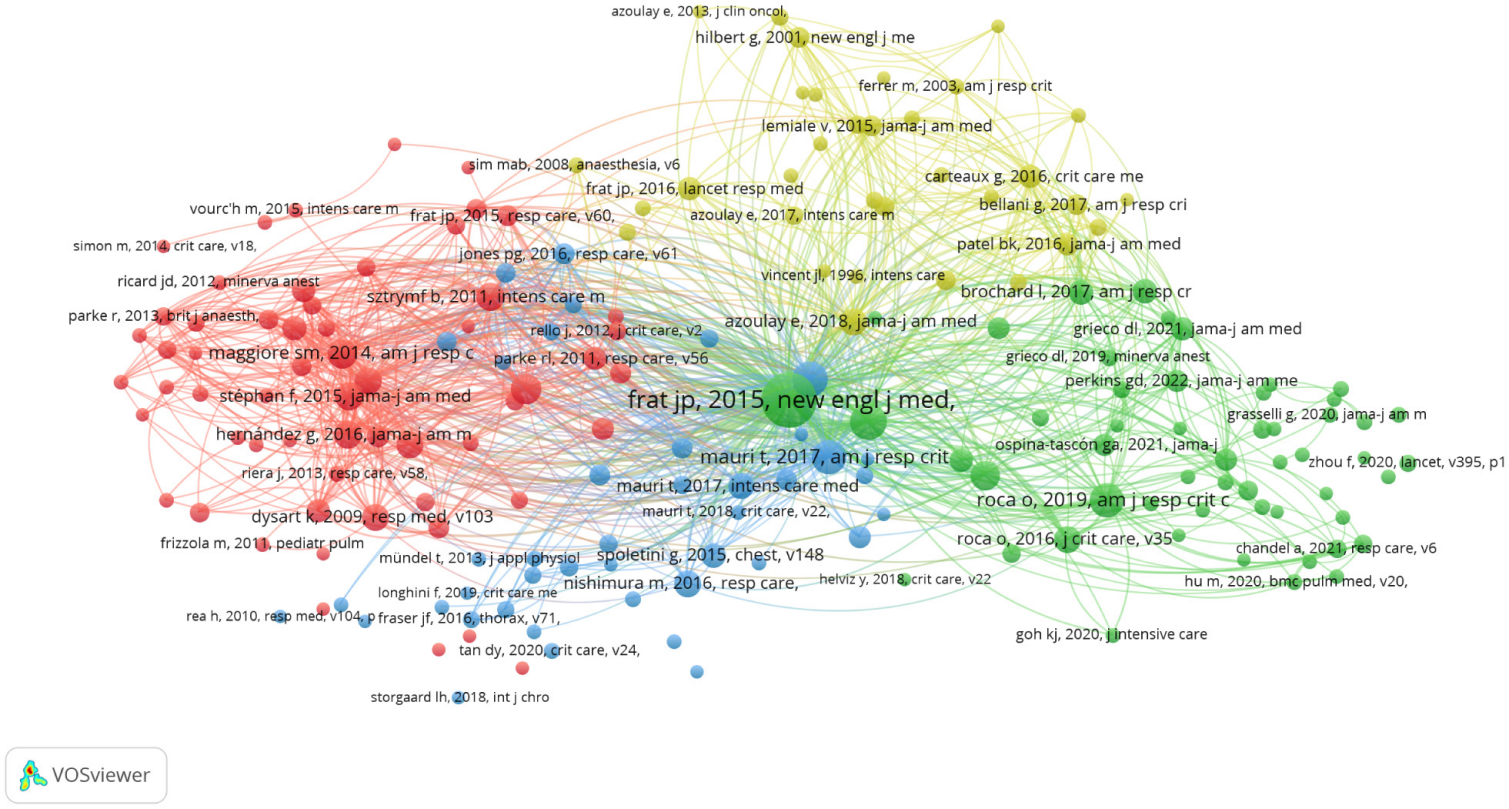
Visualization of reference co-citation analysis for HFNC applications in ARF.

Table 5 presents the top 10 co-cited references ranked by citation frequency. The most cited publication was “High-flow oxygen through nasal cannula in acute hypoxemic respiratory failure” (*New England Journal of Medicine*). To further delineate the intellectual landscape, a network visualization of the 187 most co-cited references was generated using VOSviewer (Figure 7). This mapping elucidates key citation patterns and clusters, providing valuable insights into the core knowledge structure and seminal contributions within HFNC with ARF.

**Table 5:** Leading 10 publications by citation count.

Rank	Title	Corresponding author	Journal (IF 2025)	Publication year	Total citation (*n*)
1	High-flow oxygen through nasal cannula in acute hypoxemic respiratory failure	Frat JP	*N Engl J Med* (IF 78.5)	2015	334
2	Failure of high-flow nasal cannula therapy may delay intubation and increase mortality	Kang BJ	*Intensive Care Med* (IF 21.2)	2015	147
3	Physiologic effects of high-flow nasal cannula in acute hypoxemic respiratory failure	Mauri T	*Am J Respir Crit Care Med* (IF 19.3)	2017	131
4	High-flow oxygen therapy in acute respiratory failure	Roca O	*Respir Care* (IF 2.1)	2010	105
5	Nasal high-flow vs. Venturi mask oxygen therapy after extubation: effects on oxygenation, comfort, and clinical outcome	Maggiore SM	*Am J Respir Crit Care Med* (IF 19.3)	2014	99
6	Effect of high-flow nasal oxygen vs. conventional oxygen therapy on reintubation in adults after extubation: a randomized clinical trial	Hernández G	*JAMA* (IF 55.0)	2016	92
7	Oxygen delivery through high-flow nasal cannula increase end-expiratory lung volume and reduce respiratory rate in post-cardiac surgical patients	Corley A	*Br J Anaesth* (IF 9.2)	2011	83
8	Effect of postextubation high-flow nasal cannula vs. non-invasive ventilation on reintubation and postextubation respiratory failure in high-risk patients: a randomized clinical trial	Hernández G	*JAMA* (IF 55.0)	2016	83
9	Effect of high-flow nasal oxygen vs. standard oxygen on 28-day mortality in immunocompromised patients with acute respiratory failure: the high randomized clinical trial	Azoulay E	*JAMA* (IF 55.0)	2018	81
10	Research in high flow therapy: mechanisms of action	Dysart K	*Respir Med* (IF 3.1)	2009	81

## 4 Discussion

HFNC therapy has attracted unprecedented research attention, as reflected by the steady growth in publication volumes from 2015 to 2022. It is important to note that this increasing research activity does not by itself demonstrate clinical efficacy; rather, it indicates growing scientific interest and the need for rigorous evidence synthesis. This comprehensive research reveals four critical dimensions shaping current HFNC bibliometric analysis: comparative respiratory medicine studies demonstrate established methodological frameworks, recent trends show COVID-19-accelerated evidence maturation, emerging research directions indicate technology-driven personalized approaches, and methodological considerations highlight the need for interdisciplinary analytical strategies in respiratory medicine bibliometrics. The COVID-19 pandemic fundamentally transformed respiratory care research, accelerating HFNC evidence generation by an estimated 5–7 years and establishing the therapy as a cornerstone intervention supported by multiple international guidelines (19). Current research trajectories indicate sustained growth in Artificial Intelligence (AI) integration, home care applications, and precision medicine approaches, while methodological advances in bibliometric analysis offer enhanced tools for understanding complex interdisciplinary research landscapes.

Respiratory medicine bibliometrics reveal distinct publication patterns and methodological approaches that provide crucial context for HFNC research analysis. Global research production demonstrates significant geographic concentration, with the USA contributing 37% of respiratory medicine publications and Western Europe accounting for 40.4% of output between 2007 and 2017 (20). ECMO research bibliometrics offer particularly relevant methodological insights for HFNC analysis, given their shared focus on advanced respiratory support technologies. Wang et al.'s (21) 10-year analysis of 5,986 ECMO articles using CiteSpace 5.8.R1 software identified three distinct research phases: initial clinical focus (2011–2013), diversification period (2014–2018), and COVID-19 explosion (2019–2021). This temporal framework provides a template for analyzing HFNC research evolution, particularly the pandemic's catalytic effect on respiratory support research. The methodological sophistication of respiratory medicine bibliometrics has evolved significantly, with studies increasingly employing advanced visualization tools like VOSviewer and CiteSpace for network analysis. Mechanical ventilation bibliometrics demonstrate interdisciplinary complexity (22), as shown in Ramli et al.'s (23) 60-year analysis revealing medicine's dominance but engineering and computer science integration since 1992. This multidisciplinary evolution mirrors HFNC research patterns, where clinical evidence increasingly integrates with technological innovation and AI applications.

HFNC research experienced explosive growth during 2019–2022, with publication volumes increasing from approximately 70 papers in 2019 to over 250 in 2022, representing sustained annual growth. This expansion was driven by COVID-19's urgent respiratory care needs, fundamentally altering research priorities and accelerating evidence generation processes (23). Again, the surge in publication numbers reflects heightened research focus and expedited knowledge dissemination, not an inherent confirmation of HFNC's superiority (24). The pandemic catalyzed three distinct phases of HFNC research evolution: emergency application studies (2020–2021), systematic consolidation (2022–2023), and optimization and diversification (2024-present). COVID-19 studies established crucial predictive tools, particularly the ROX index (SpO_2_/FiO_2_/RR), with ROX >5.55 at 6 h post-initiation showing strong association with HFNC success (19) (OR 17.821) across multiple validation studies (25). Clinical guidelines maturation represents a critical milestone in HFNC research evolution. The European Respiratory Society 2022 guidelines provided conditional recommendations for HFNC over conventional oxygen therapy in hypoxemic acute respiratory failure (2), while 2023 ESICM ARDS guidelines offered strong recommendations for HFNC over conventional oxygen (26). These guideline developments transformed HFNC from experimental therapy to evidence-based standard of care, fundamentally shifting research focus from efficacy to optimization.

AI applications in respiratory care are gaining attention, though evidence specific to HFNC remains preliminary. Recent reviews suggest potential roles for AI in predicting HFNC failure, optimizing flow settings, and monitoring treatment response, but most models have not yet undergone prospective validation in HFNC cohorts (27, 28). It is projected that future respiratory devices may incorporate AI-driven decision support, but current clinical application remains investigational (29). Therefore, while AI holds promise for personalizing HFNC therapy, readers should interpret existing findings cautiously, as most derive from heterogenous patient populations or simulated environments. Research focus during 2020–2021 centered on COVID-19 respiratory support, shifting toward chronic disease and technological innovation by 2024 (30). This progression indicates field maturation and diversification beyond acute care applications toward chronic disease management and home care implementation.

Clinical applications expansion represents the most immediate research opportunity, with home HFNC therapy showing particular promise for chronic respiratory diseases. Systematic review evidence demonstrates that home HFNC reduces exacerbations and improves quality of life in COPD and bronchiectasis patients (31). Emerging evidence also supports the use of HFNC during exercise training and pulmonary rehabilitation in chronic respiratory diseases, with improvements in exercise endurance and dyspnea scores (32, 33). Post-operative care applications and palliative care settings represent additional growth areas with emerging evidence bases (34). Methodological improvements in bibliometric analysis offer enhanced analytical capabilities for future HFNC research assessment. The transition toward sensemaking approaches beyond descriptive statistics, integration of alternative databases like Dimensions (35), and advanced visualization using machine learning-enhanced network analysis provide more sophisticated tools for understanding research landscapes (36). These methodological advances enable more nuanced interpretation of HFNC research evolution and impact assessment.

Personalized medicine integration represents a paradigmatic shift in respiratory care research, with HFNC positioned to benefit from precision medicine approaches. Development of patient subphenotypes in ARDS using genomic, proteomic, and metabolomic techniques (37), combined with biomarker-guided therapy (such as blood eosinophil levels >300 cells/ml predicting inhaled corticosteroid response) (38), suggests future HFNC research will increasingly focus on patient-specific optimization rather than one-size-fits-all approaches. AI and machine learning applications demonstrate remarkable potential for transforming HFNC clinical decision-making. Current achievements include AI software achieving 82% correct diagnosis vs. 44.6% for pulmonologists in pulmonary function test interpretation, and machine learning models reaching 94% accuracy in COPD detection from pulmonary audio data (39, 40). Future applications include predictive models for HFNC failure, automated monitoring systems, and personalized therapy optimization.

Alternative metrics integration offers enhanced analytical capabilities for HFNC research assessment, capturing broader impact through social media mentions, news coverage, policy document citations, and download statistics (41). These altmetrics provide immediate impact assessment and broader audience capture beyond academic communities, particularly valuable for clinical innovations like HFNC therapy with significant public health implications (42). Network analysis advances enable sophisticated collaboration and knowledge transfer assessment, using co-citation networks for intellectual structure mapping and author collaboration networks for research community analysis (43). These approaches are particularly valuable for understanding international collaboration patterns in respiratory medicine and identifying emerging research communities around specific HFNC applications. Machine learning applications in bibliometrics offer automated paper classification, citation prediction, and research trend identification capabilities (44). Natural language processing enables content analysis, while deep learning approaches enhance citation context understanding. These emerging methodologies provide scalable solutions for analyzing the rapidly expanding HFNC research corpus.

Future HFNC bibliometric research should prioritize multi-database approaches to ensure comprehensive coverage, implement field-specific normalization techniques for respiratory medicine, and integrate traditional and alternative metrics for holistic impact assessment. The interdisciplinary nature of HFNC research requires sophisticated analytical approaches that account for clinical, technological, and policy dimensions of research impact (45). Quality assessment remains paramount, requiring transparent methodology documentation, expert validation processes, and attention to potential biases. As HFNC research continues expanding into chronic care, home applications, and AI-enhanced systems (46–48), bibliometric analysis must evolve to capture these emerging dimensions of research impact and clinical translation effectiveness.

Although extensively employed in the academic community, bibliometrics has notable limitations as a tool for assessing research and academic output. Firstly, our analysis was restricted to publications indexed in WOSCC, PubMed and Scopus, potentially excluding relevant studies from other databases or repositories. For respiratory medicine research, PubMed offers specialized MeSH optimization and comprehensive life sciences coverage (49), Scopus provides multidisciplinary breadth, and Web of Science delivers highest quality citation network data (50). Secondly, interdisciplinary complexity poses particular challenges for HFNC bibliometric analysis, as respiratory medicine research spans pulmonology, critical care, immunology, and environmental health. Furthermore, temporal limitations affect HFNC research assessment, as papers require minimum 2–3 years to accumulate reliable citation counts. This creates systematic bias toward older publications and may undervalue recent innovations in HFNC therapy. Despite these drawbacks, bibliometrics remains a valuable tool for scholars to quickly identify research hotspots and development trends, thereby facilitating further investigation.

## 5 Conclusion

This comprehensive analysis reveals that HFNC research has rapidly expanded in volume, reflecting intense scientific interest and accelerated evidence generation during the COVID-19 pandemic. However, publication trends alone should not be interpreted as proof of clinical effectiveness; rigorous clinical trials and guideline syntheses remain the gold standard for establishing therapeutic value. The field's transition from experimental therapy to evidence-based standard of care represents one of respiratory medicine's most significant recent advances, supported by robust international guidelines and sustained research investment. Future HFNC bibliometric research should emphasize methodological rigor through multi-database integration, interdisciplinary analytical approaches, and emerging metrics incorporation to fully capture the field's complexity and clinical impact. As HFNC therapy expands into personalized medicine, AI-enhanced systems, and chronic care applications, bibliometric analysis must evolve to support evidence-based decision-making in this critical area of respiratory medicine.

## Data Availability

The original contributions presented in the study are included in the article/supplementary material, further inquiries can be directed to the corresponding authors.
